# Design, synthesis, and study of Pd(II) ion‐imprinted functionalized polymer

**DOI:** 10.1002/ansa.202000030

**Published:** 2020-06-25

**Authors:** Mohammad Nozari, Mohammed Monier, Ali H. Bashal

**Affiliations:** ^1^ Department of Chemistry Drexel University Philadelphia Pennsylvania USA; ^2^ Chemistry Department Faculty of Science Mansoura University Mansoura Egypt; ^3^ Chemistry Department Faculty of Science Taibah University Yanbu ElBahr KSA

**Keywords:** (3‐aminopropyl)triethoxysilane, ion‐imprinting, isatin, palladium ions, selective extraction

## Abstract

Selective metal ions’ extraction and recovery has various applications in the analytical field. Metal ions need to be extracted, detected, and quantified. For that purpose, ion‐imprinted polymers have earned a great deal of attention during the past two decades. Pd^2+^ ion‐imprinted hollow silica particles including an isatin Schiff base were prepared by Schiff base condensation of (3‐aminopropyl)triethoxysilane and isatin. The prepared Schiff base ligand was coordinated to the target Pd^2+^ cations, the polymerizable Pd‐complex was set aside to form gel in the company of tetraethoxysilane and the target Pd^2+^ cations were subsequently removed from the cross‐linked silica network by means of acidified thiourea solution. All materials throughout this synthesis process were investigated utilizing mass spectrometry, elemental analysis, FTIR, and ^1^H‐NMR. The morphological structure of both Pd^2+^ ion‐imprinted and non‐ion‐imprinted silica polymer were pictured by scanning electron microscopy. Several batches were studied exploiting both Pd^2+^ ion‐imprinted and non‐ion‐imprinted silica polymer to test their functionality for selective extraction of Pd^2+^ cations in multi‐ionic solution of Ni^2+^, Co^2+^, Cu^2+^, Mn^2+^, and Pd^2+^.

## 1 INTRODUCTION

The particular chemical, physical, and biological characteristics of precious metals such as platinum‐group metals (PGMs), which include platinum (Pt), palladium (Pd), iridium (Ir), osmium (Os), rhodium (Rh), ruthenium (Ru) as well as gold (Au) and even silver (Ag), have attracted a great deal of attention in various fields of application such as catalysis, renewable energy systems, and the electronic and pharmaceuticals industries.^1^, ^2^, ^3^, ^4^, ^5^ Within the next few years, the expected high demand for these metals and the depletion of them in the earth's crust will escalate their price. Therefore, the extraction and retrieval of these metal ions from industrial wastewater gains extreme economic importance.^6^

Co‐precipitation,^7^, ^8^ solid phase extraction,^9^ solvent extraction,^10^ membrane ultra‐filtration, and reverse osmosis^11^, ^12^ are well‐known techniques, which have been commonly utilized in separation of heavy metal ions from aquatic wastes. However, the wide utilization of these methods is greatly restricted due to the high cost, energy demand, low separation efficiency and formation of harmful and sometimes toxic by‐products, which could be released, causing soil, or water pollution.^13^, ^14^ Adsorption is regarded as one of the most efficient known techniques, which is able to extract heavy metal cations from aqueous solutions without the drawbacks of the previously mentioned methods.^15^ In previous studies, various adsorbents were prepared and utilized for removal of different metal ions such as modified grafted cellulosic cotton fibers,^16^, ^17^ grafted PET fibers,^18^ modified chitosan, and alginate.^19^, ^20^, ^21^, ^22^ Despite the obvious competence of these adsorbents, the absence of selectivity could be a serious limitation to their use on a large scale.

Ion‐imprinting is considered a relatively modern technique utilized for improving the adsorbent selectivity toward target specific ionic species and rare earth metals in the environment mixed with other interfering ions.^23^, ^24^ The common method for the preparation of ion‐imprinted polymeric materials includes complex formation between the target ionic species and an active polymerizable ligand followed by polymerization in presence of an appropriate cross‐linker agent. The metal ions are then extracted from the cross‐linked polymeric network to leave recognition sites capable of selectively interacting with the same metal ions over the other interfering ones.^25^, ^26^, ^27^, ^28^, ^29^

As a result of its high rigidity, which is considered a vital point in creation of active recognition sites, the imprinted matrices derived from mesoporous silica are regarded as one of the most promising materials. Moreover, these materials provide the target species with a high accessibility to the active recognition sites, due to the relatively high pore volume and nano‐scaled pore wall, particularly with denser cross‐linked structures, which limit the molecules’ mobility. Generally, the functionalized polymeric matrices based on mesoporous silica can be obtained by co‐condensation of tetraalkylorthosilicate in the presence of other organosilica precursors, depending on the desired functional group.

In the literature, there are several material used to create cross‐linked network for ion‐imprinting, for instance, Francisco *et al* ^30^ used 4‐vinylpyridine as polymerizable agent, ethylene glycol dimethacrylate as the crosslinking agent, and benzoyl peroxide for radical generator. Kumar *et al* ^31^ and da Santos Silva *et al* ^32^ utilized 1‐vinyl imidazole as ligand and methacrylic acid as functional monomer for synthesis of ion‐imprinted polymer. Biswas *et al* ^33^ used modified curcumin as functional monomer, ethylene glycol dimethacrylate as a binding agent and 2,2‐azobisisobutyronitrile as a free radical initiator. Yasizai *et al* ^34^ reported ion‐imprinted polymer based receptors based on styrene, N‐vinylpyrrolidone, and their copolymer. Table 1 shows a comparative review of different methodologies for detection and recovery of the palladium in aqueous samples. Several factors need to be considered for designing novel IIPs such as maximum absorbance capacity (*q_m_*), pH of application, recovery percentage, ease of synthesis, and affordability. Therefore, it needs to be highlighted that based on comparative data in Table 1, we propose an easy to synthesize, accessible alternative that offers the second highest maximum absorbance capacity for palladium 249 mg/g; one should note that the previous work^38^ that has higher absorbance capacity 275 mg/g has a lower recovery percentage 65% compared to our proposed method 98%, which overall makes the proposed Pd‐IIP a method that offers better recovery and quantification results.

**TABLE 1:** Different methodologies for selective palladium detection in water samples

Reference	Methodology	Detection	pH	q_m_ ^*^ (mg/g)	Recovery (%)
Fujiwara et al 2007^20^	l‐Lysine modified chitosan IIP	ICP‐AES^a^	2	109	98
Godlewska‐Żyłkiewicz et al 2010^35^	4‐vinylpyridin & styrene IIP	FAAS^b^	5‐6	13.3	92‐100
Liu et al 2012^15^	chitosan & graphene oxide composite	FAAS	3‐4	216	95
Jiang et al 2013^36^	2‐aminobenzonitrile & 4‐vinylpyridin IIP	AAS^c^	0.5	38.9	–
Cataldo et al 2014^37^	modified alginate beads	ICP‐OES^d^	3	127	–
Lin et al 2015^2^	chitosan ion‐imprinted fiber	ICP‐AES	2	174	96
Monier et al 2016^38^	modified chitosan resin	ICP‐AES	6	275	65
Yu et al 2018^39^	2‐hydroxyethyl methacylate IIP	AAS	3‐4	46	–
Present Work	silane & isatin IIP	ICP‐AES	5‐6	249	98

* q_m_ maximum absorbance capacity.

a ICP‐AES (Inductively coupled plasma ‐ atomic emission spectrometer).

b Flame atomic absorption spectrometry).

c Atomic absorption spectrum).

d ICP‐OES (Inductively coupled plasma ‐ optical emission spectrometry).

Herein, we report the synthesis and full characterization of Pd^2+^ ion‐imprinted hollow silicone particles (Pd‐Si‐IS). The proposed Schiff base precursor ligand AT‐IS was initially produced by reaction of (3‐aminopropyl)triethoxysilane (AT) with isatin (IS). Then the template Pd(II) ions were complexed to the AT‐IS ligand and the polymerizable Pd‐complex obtained was set aside to form gel in with tetraethoxysilane (TEOS). Afterward, the Pd^2+^ ion‐imprinted silica network was prepared through mechanically smashing the resulting solid matter followed by Pd^2+^ ion desorption. All materials prepared throughout this synthetic process were investigated using elemental analysis, FTIR, and scanning electron microscopy (SEM). In addition, both the ion‐imprinted and the non‐ion‐imprinted silica particles were tested for their functionality in selective extraction of Pd^2+^ ions under various interfering effects of accompanying ions in order to evaluate the optimal factors that affect the selective extraction.

## 2 MATERIALS AND METHODS

### 2.1 Chemicals

(3‐Aminopropyl)triethoxysilane (AT), isatin (IS), tetraethoxysilane (TEOS), and PdCl_2_ were purchased from Alfa Aesar Chemicals and were utilized without any additional treatment. All utilized solvents and chemicals were obtained from Sigma–Aldrich and utilized as received.

### 2.2 Synthesis of HATIS Schiff base ligand

Initially (35 mmol, 5 g) of isatin was dissolved in 100 mL 200 proof anhydrous ethyl alcohol to give a clear solution, to which AT (35 mmole, 7.53 g) was added. The system was then refluxed for 4 h, after which the reaction mixture was cooled and AT‐IS Schiff base precipitated by addition of ether, the ligand was filtered off and washed numerous times with 200 proof anhydrous ethyl alcohol.

### 2.3 Preparation of the monomer‐template Pd^2+^ complex

AT‐IS Schiff base (40 mmol, 14 g) was dissolved in 100 mL hot anhydrous ethyl alcohol to which (20 mmol, 3.57 g) PdCl_2_ was then added. The reaction container was connected to a reflux condenser and the temperature raised to 80°C for 6 h with continuous magnetic stirring. The mixture was then cooled and the monomer‐template Pd‐complex was filtered off then washed with anhydrous ethyl alcohol.

### 2.4 Preparation of Pd^2+^ ion‐imprinted silica particles (Pd‐Si‐IS)

**FIGURE 1:**
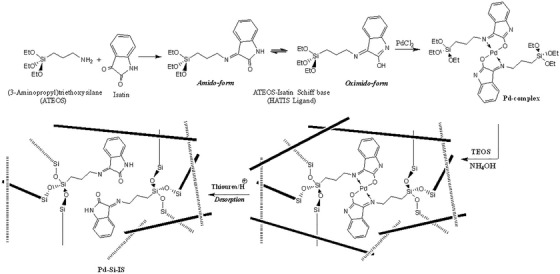
Preparation of Pd^2+^ ion‐imprinted silica particles

Typically, (7.5 mmol, 6.5 g) of Pd‐complex monomer template was dissolved in 15 mL TEOS. Then the NH_4_OH (1 M, 3 mL) mixed with 5 mL ethanol was added dropwise over in 15 min with continuous mixing. The mixture obtained was set aside in a desiccator for 48 h at ambient temperature and then dried in oven at 40°C for 24 h. The resulting particles were collected, mechanically ground to approximately 200 μm particle size, then washed continuously with ethyl alcohol, diluted HCl, and distilled water. Afterward, the Pd^2+^ cations were taken out of the cross‐linked network by stirring the silica particles in 300 mL 0.5 M acidified thiourea solution until all Pd^2+^ ions were entirely removed. Then Pd‐Si‐IS particles were washed with distilled water until remaining acid was removed, and then dried in the oven for 12 h at 40°C. For selectivity comparison, a blank non‐ion‐imprinted silica network was prepared by following the same procedure by AT‐IS Schiff base ligand instead of the Pd‐complex. The synthetic procedures are displayed in Figure 1.

### 2.5 Instrumentation

A Perkin–Elmer 240C Elemental Analytical Instrument was utilized in performing C, H, and N elemental analyses. Palladium and chloride were estimated using the digestion method.^40^ Infrared spectroscopy was completed on a Perkin–Elmer spectrometer. NMR spectra were obtained on Oxford 500 MHz instrument with samples in DMSO‐d_6_. Morphological structures were studied by scanning electron microscope (FEI Co.). The mass spectra were obtained by Agilent‐7800 ICP‐MS. Surface area measurement was performed by BET technique using a Micromeritics ASAP 2010 instrument. The amounts of cations were measured the using Inductively Coupled Plasma‐Atomic Emission Spectrometer (ICP‐AES; ICPS‐7500, Shimadzu, Japan).

### 2.6 Batch adsorption studies

For preparing the batches, 50 mg of either Pd‐Si‐IS or NII‐Si‐IS particles were immersed in 50 mL Pd^2+^ cation solution with known concentrations. The solution pH was altered to the determined values by means of the proper buffer solutions. The containers were located on a thermostated shaker adjusted to 150 rpm at the desired temperatures for appropriate periods of time (10‐120 min). After the suspensions reached equilibrium, the containers were taken out of the shaker, the particles were filtered off and the amounts of adsorbed Pd^2+^ cations were determined by measuring the residual amount in the filtrate using inductively coupled plasma‐atomic emission spectrometer (ICP‐AES) and using the Equation (1).

(1) $$q_{e}=\left(C_{i}-C_{e}\right)V/W$$

where *q_e_* (mg/g) is the adsorbed Pd^2+^ at equilibrium; *C_i_* (mg/L) and *C*_e_ (mg/L) are the initial and final Pd^2+^ cation concentrations, respectively, *V* (L) is solution volume, and *W*(g) is the particles’ weight.

### 2.7 Initial pH effect

The Pd^2+^ adsorption using Pd‐Si‐IS and NII‐Si‐IS was studied in the pH ranging 1‐6 along with 50 mg of the target adsorbent particles in 50 mL of 200 ppm aqueous Pd^2+^ cations solution at 30°C. The batches were equilibrated on a shaker at 150 rpm for 3 h then filtered to calculate the residual metal ion amount. The removal percentage was calculated by applying Equation (2).

(2) $$\mathrm{Percent}\;\mathrm{removal}\;\left(\%\right)=\frac{\left(C_{i}-C_{e}\right)}{C_{i}}\times \;100$$

### 2.8 Effect of temperature

A series of batches containing 50 mg of the target adsorbent particles immersed in 50 mL of 50 ppm aqueous Pd^2+^ cation solution was equilibrated in the temperature ranging from 20 to 40°C. After the reaction was complete the residual Pd^2+^ contents were estimated as previously described.

### 2.9 Kinetics

Two sets were made ready, each by soaking 500 mg of either Pd‐Si‐IS or NII‐Si‐IS in 500 mL of 200 ppm Pd^2+^ cation solution. The containers were equilibrated at 30°C and 150 rpm and pH 5, an aliquot of 1 mL was sampled every 10 min to evaluate the level of Pd^2+^ cation residue in the solution.

### 2.10 Isotherm experiments

Several batch adsorption tests were completed using initial adsorbate Pd^2+^ cations for which the concentration was changed from 50 to 500 ppm. In each instance, either Pd^2+^ ion‐imprinted or non‐ion‐imprinted silica particles (50 mg) were equilibrated in 50 mL aqueous Pd^2+^ cation solution at 150 rpm and 30°C for 3 h.

### 2.11 Ion selectivity

For evaluating the effect of the ion‐imprinting process on improving the selectivity of the silica particle for the target Pd^2+^ cations, the extraction process was completed in a solution containing 40 ppm of several metal ions of the interfering ions Co^2+^, Cu^2+^, Mn^2+^, and Ni^2+^ together with the target Pd^2+^ cations using either Pd‐Si‐IS or NII‐Si‐IS silica particles. After agitating at 150 rpm, pH 5, and 30°C for 3 h, each set was filtered in order to measure the residual amounts of Pd^2+^ cations and each co‐existing interfering metal cation. The following relationship was used to evaluate the imprinting effect on the selective extraction of Pd^2+^ cations.^41^

(3) $$D=\frac{\left(C_{i}-C_{f}\right)}{C_{f}}\times \frac{V}{W}$$

where *D*, *C_i_*, and *C_f_* (mg/L) are the distribution coefficient and initial and final Pd^2+^ concentrations, respectively.

The affinity of each of the investigated silica particle types toward Pd^2+^ was contrasted to interfering metal ions was using:

(4) $$\beta_{Pd^{2+}/M^{n+}}=\frac{D_{Pd^{2+}}}{D_{M^{n+}}}$$

While the impact of the ion‐imprinting could be estimated by using Equation (5):

(5) $$\beta_{r}=\frac{\beta_{imprinted}}{\beta_{non-imprint}}$$

### 2.12 Regeneration

A mixture solution of 1:1 aqueous 1 M thiourea and 1 M acid (HCl) was used for desorption and regeneration. The Pd^2+^ cation‐loaded silica particles were agitated with this regeneration solution for 2 h at 150 rpm at ambient temperature. The particles were then extracted and treated with dilute NaOH solution for neutralization and washed with distilled water before being reused in a subsequent extraction process. Desorption ratio and regeneration efficiency were calculated by:

(6) $$\mathrm{Desorption}\;\%=\left(\frac{\mathrm{Amount}\;\mathrm{of}\;\mathrm{Pd}\left(\mathrm{II}\right)\;\mathrm{desorbed}}{\mathrm{Amount}\;\mathrm{of}\;\mathrm{Pd}\left(\mathrm{II}\right)\;\mathrm{adsorbed}}\right)\times 100$$

(7) $$\mathrm{Regeneration}\;\mathrm{efficiency}\;\left(\%\right)=\left(\frac{\mathrm{Pd}\left(\mathrm{II}\right)\;\mathrm{adsorbed}\;\mathrm{in}\;\mathrm{the}\;\mathrm{second}\;\mathrm{time}}{\mathrm{Pd}\left(\mathrm{II}\right)\;\mathrm{adsorbed}\;\mathrm{in}\;\mathrm{the}\;\mathrm{first}\;\mathrm{time}}\right)\times 100$$

## 3 RESULTS AND DISCUSSION

### 3.1 Characterization

The percentage amount of C, H, and N were acquired from elemental analysis for both Pd and Cl, together with the values that were estimated by standard digestion method^40^ are collected in Table 2. The mass spectra of the Schiff base ligand HATIS presented a major signal at *m/z* 351.21 (M+1)^+^ without the characteristic signals at *m/z* 147.13 or 222.13 due to isatin or AT, respectively. These obtained results endorse the HATIS Schiff base synthesis. The mass spectra of the Pd‐complex revealed the major signal at *m/z* 806.22, as a solid evidence for complex synthesis based on the proposed composition [Pd(ATIS)_2_] (calculated *m/z* 806.22).

**TABLE 2:** Elemental analysis of HATIS and [Pd(ATIS)_2_]

	Found (calculated) (%)			
Compound	C	H	N	Metal
HATIS	58.5 (58.3)	7.4 (7.5)	7.9 (8.0)	–
[Pd(ATIS)_2_]	50.4 (50.7)	6.1 (6.3)	6.8 (7.0)	13.0 (13.2)

The Pd^2+^’s coordination to the HATIS ligand was further evidenced through comparison of the FTIR spectra of the free ligand and corresponding Pd‐complex. Table 3 summarizes the basic characteristic absorptions, which undergo the main shifts or changes upon coordination. It has been reported that isatin motifs demonstrate amido‐oximido tautomerism in solution.^42^ The synthesized ligand exhibited characteristic bands at 3244, 1720, and 1640 cm^−1^ related to N‐H, amidic C=O, and C=N, respectively, confirming that in the solid state, the ligand exists mainly in amido‐form. After coordinating the ligand to the Pd^2+^ cations, the characteristic C=N absorption exhibited a noticeable shift to 1620 cm^−1^. On the other hand, the absence of both amidic C=O and N‐H absorptions with the simultaneous emergence of a novel C=N absorption at 1650 cm^−1^ is clear evidence for the amido‐oximido transformation associated with Pd^2+^ ion‐binding and coordination via the oxygen of the deprotonated hydroxyl group and the nitrogen of the C=N species, in a square‐planar five‐membered ring system as displayed in Figure 1.

**TABLE 3:** IR absorptions (cm^−^ ^1^) of the HATIS and [Pd(ATIS)_2_]

Compound	*v*(N‐H)	*v*(C = O)	*v*(C = N)_1_	*v*(C = N)_2_
HATIS	3244	1720	1640	–
[Pd(ATIS)_2_]	–	–	1620	1650

The electronic spectra of the synthesized Pd‐complex displayed two bands at 17 300 and 19 200 cm^−1^ assigned to the ^1^A_1g_ → ^1^A_2g_ and ^1^A_1g_ → ^1^B_1g_ transitions, respectively, in square planar geometry.^43^, ^44^

**FIGURE 2:**
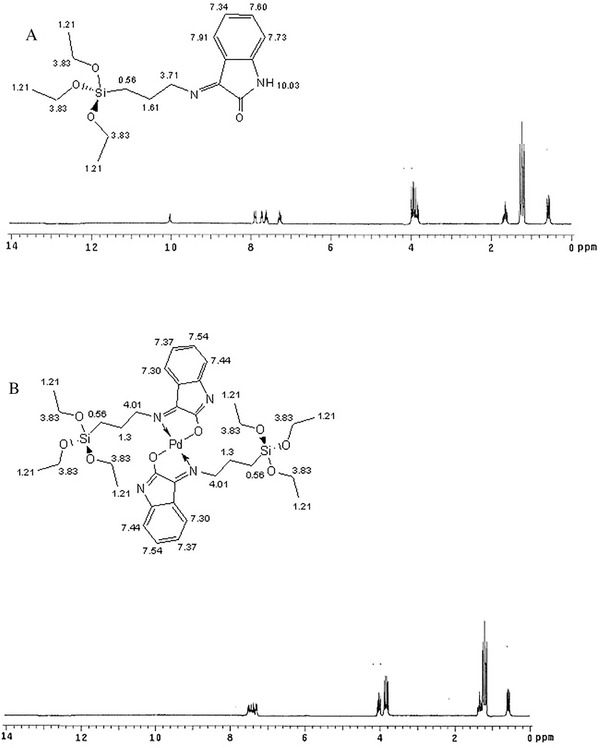
^1^H NMR spectra of (A) HATIS, (B) [Pd(ATIS)_2_]

The ^1^H‐NMR spectrum of HATIS metal‐free ligand is shown in Figure 2A, similar to previous literature,^45^ the diagnostic AT resonances were evidently noticed between 0.56 and 3.83 ppm. Another determining factor for Schiff base synthesis was the amide N‐H peak presence at 10.03 ppm, along with aromatic doublets at 7.73 and 7.93 ppm, and triplets at 7.34‐7.60 ppm, which are typical for the inserted isatin moieties.^46^ The Pd‐complex exhibited a similar spectrum (Figure 2B). However, the absence of the amidic N‐H peak indicates the Pd^2+^ coordination through the previously mentioned deprotonated oximido‐form.

**FIGURE 3:**
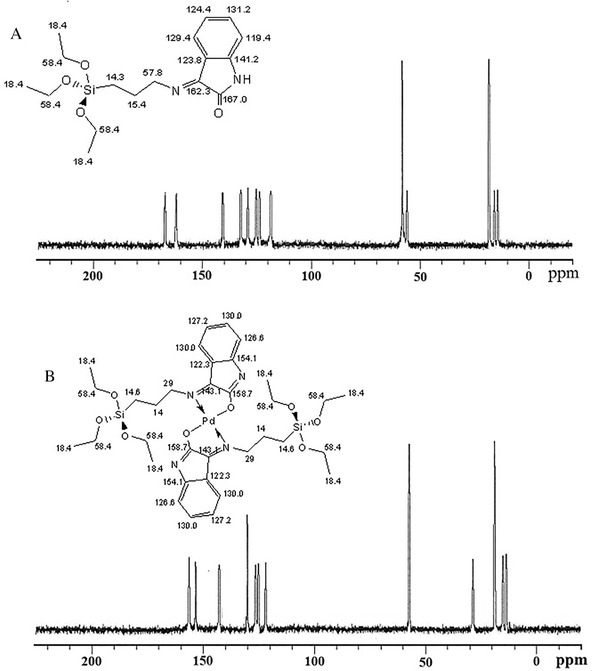
^13^C NMR spectra of (A) HATIS, (B) [Pd(ATIS)_2_]

The ^13^C‐NMR spectra of HATIS and related Pd‐complex are shown in Figure 3; the peaks for both C=N and C–O showed noticeable shifts after bonding to the Pd^2+^ cations, ratifying the involvement of these functional groups in the process of complex synthesis.

**FIGURE 4:**
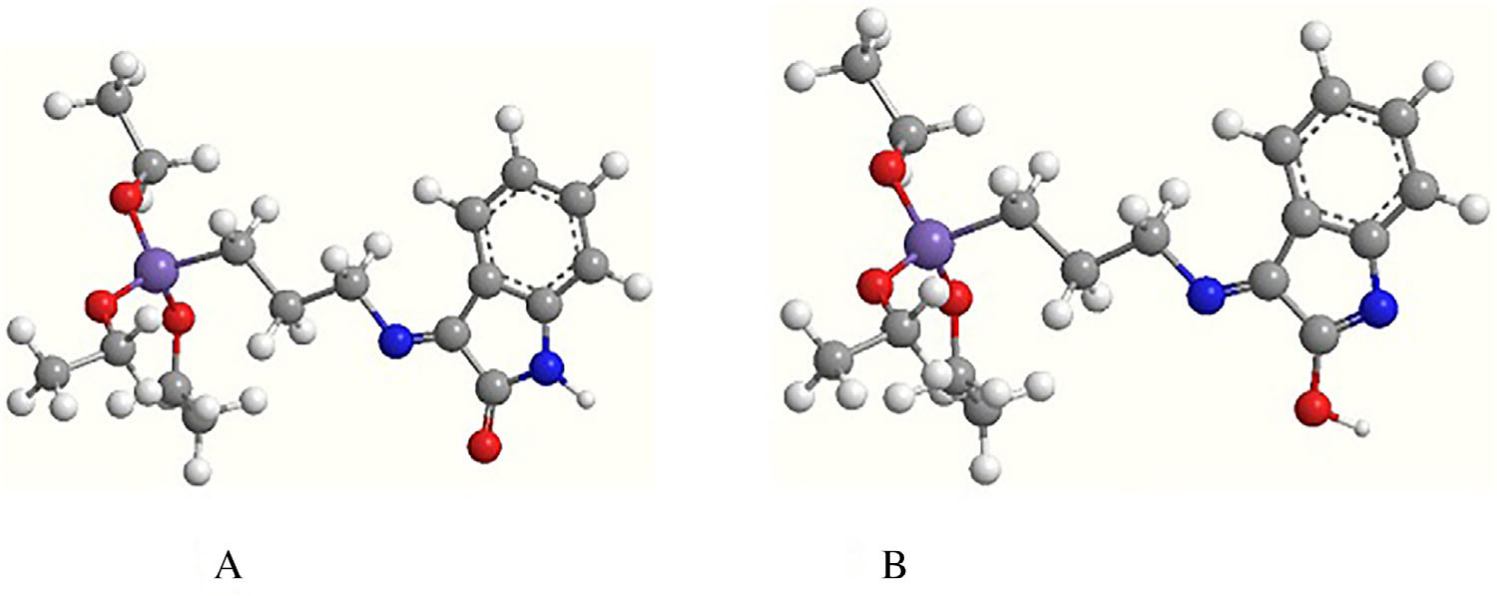
Proposed structures of (A) amido form and (B) oximido form

Conformational analysis along with geometry optimization for both HATIS ligand and Pd‐complex were performed by HyperChem 8.0 software and utilizing the MM+ and semi‐empirical PM3 force‐field methods. Molecular models of the ligand in both amido and oximido tautomeric forms are presented in Figure 4. The calculated theoretical total energies were 20.768 and 27.287 kcal/mol for amido and oximido forms, respectively. These obtained results are in line with the experimental results from the FTIR spectra, which confirm the existence of the amido rather than oximido form in solid state.

**FIGURE 5:**
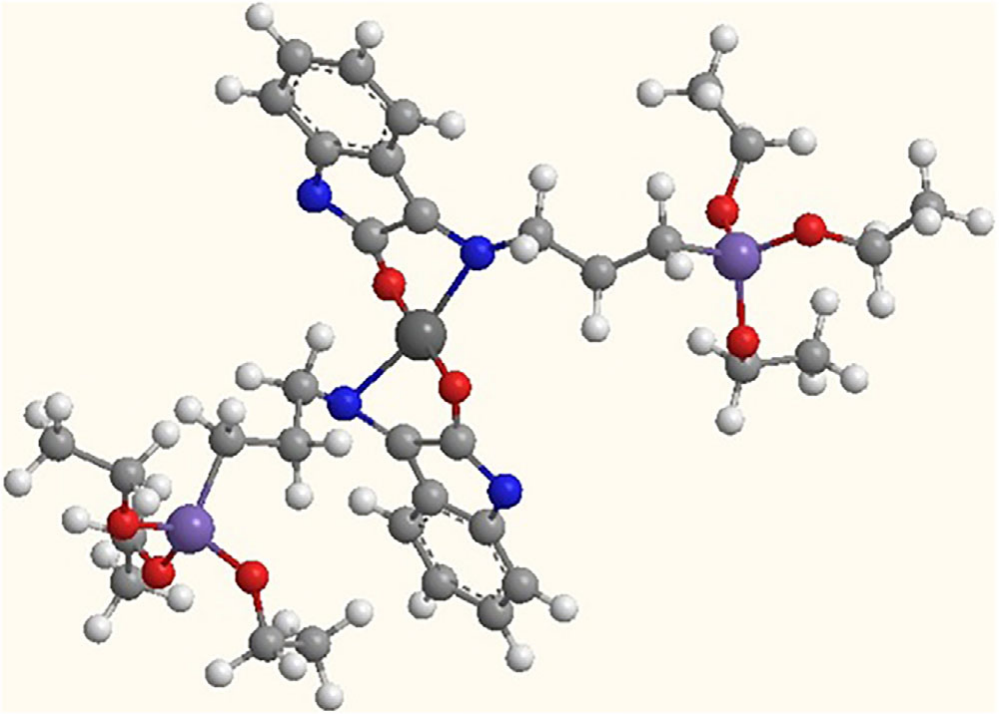
Molecular model of Pd‐complex

The proposed geometry‐optimized structures of the Pd‐complex are presented in Figure 5, the square planar geometry of the Pd‐complex provides proper organization of the EtO groups with the least steric interruption for an effective sol‐gel polymerization.

**FIGURE 6:**
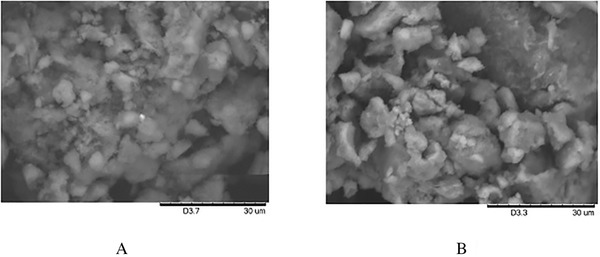
SEM images of (A) Pd‐Si‐IS and (B) NII‐Si‐IS

The surface morphology of both ion‐imprinted and non‐ion‐imprinted silica particles was pictured by SEM (Figure 6). The ion‐imprinted Pd‐Si‐IS exhibited a rough surface compared to the smooth one observed for non‐ion‐imprinted NII‐Si‐IS. The resultant rough hollow morphology may be attributed to the Pd^2+^ extraction from the cross‐linked matrix of the silica particles. Moreover, the surface area obtained by BET measurements exposed a rough irregular morphology as a result of the ion‐imprinting. Pd‐Si‐IS and NII‐Si‐IS exhibited areas of 1220.25 and 895.45 m^2^/g, respectively.

**FIGURE 7:**
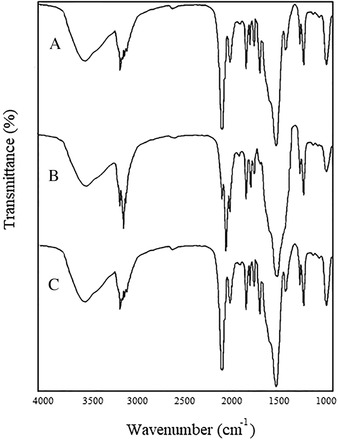
FTIR spectra of (A) NII‐Si‐IS, (B) Pd‐Si‐IS before Pd(II) ion removal, and (C) Pd‐Si‐IS after Pd^2+^ ion removal

The FTIR spectra for both Pd^2+^ ion‐free and Pd‐loaded Pd‐Si‐IS along with the NII‐Si‐IS are shown in Figure 7; both the Pd^2+^ ion‐free Pd‐Si‐IS and NII‐Si‐IS particles gave approximately identical spectra with characteristic IR absorptions at 3244, 1720, and 1640 cm^−1^ related to N‐H, amidic C=O, and C=N, respectively. These observations endorse that the active isatin Schiff base species are still included in the cross‐linked silica network after the Pd^2+^ cation leaching treatment. On the other hand, the Pd^2+^ ion‐loaded Pd‐Si‐IS particles demonstrated spectra close to the one observed for the Pd‐complex, which indicates that bonding to Pd^2+^ ions induces the amido‐oximido transformation and coordination via the oxygen of the deprotonated hydroxyl group and the nitrogen of the C=N species in a square‐planar five‐membered ring system.

### 3.2 Adsorption studies

#### 3.2.1 Solution pH effect

**FIGURE 8:**
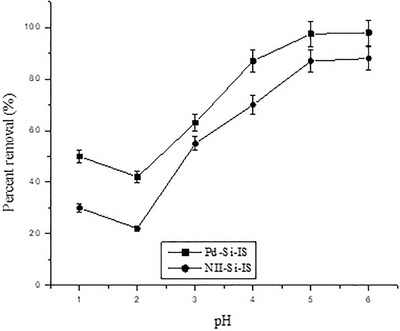
Effect of pH on the removal of Pd^2+^ ions by Pd‐Si‐IS and NII‐Si‐IS

The adsorption of ionic species in aqueous solutions is typically distressed by the solution pH, which determines the charge and surface ionization. The percentage removal of Pd^2+^ cations by Pd‐Si‐IS and NII‐Si‐IS were determined for the range 1 ≤ pH ≤ 6 (Figure 8). At pH 1, the percentage removal was moderately elevated that can be attributed to the ionic interactions between the dominant [PdCl_4_]^−^ anions and the protonated functional groups on the adsorbent surface. Conversely, the adsorption reduced at pH 2. By increasing pH, both adsorbents exhibited greater removal efficacy. In low acidic media, palladium ions exist generally as divalent Pd^2+^ cations, conversely the chelating sites still have positive charges. This phenomenon can in turn limit the target Pd^2+^ metal ions’ approaching to the active sites.

#### 3.2.2 Thermodynamics

To understand the adsorption systems’ spontaneity, it is useful to determine the thermodynamic parameters. The adsorption of Pd^2+^ cations by both Pd‐Si‐IS and NII‐Si‐IS was thus performed at various temperatures and the results were used to calculate the standard free energy (Δ*G*°), enthalpy (Δ*H°*), and entropy (Δ*S*°) values for adsorption, employing the Van't Hoff approach.^47^

(8) $$lnK_{d}=-\frac{\Delta G^{0}}{RT}=\frac{\Delta S^{0}}{R}-\frac{\Delta H^{0}}{RT}$$

**FIGURE 9:**
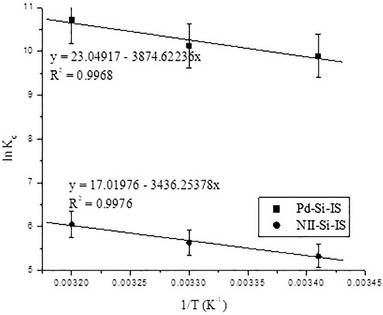
LnK_C_ plot for the Pd^2+^ cations’ uptake by Pd‐Si‐IS and NII‐Si‐IS chelating silica particles

Estimates for ΔH° and *ΔS°* were obtained as shown in Figure 9.

*ΔG°_ads_*, *ΔH°_ads_* and *ΔS°_ads_* values for Pd^2+^ extraction for Pd‐Si‐IS and NII‐Si‐IS are displayed in Table 4. In all of the tested temperatures, the adsorption process was spontaneous. Also, Pd^2+^ ion adsorption is enthalpically disfavored but entropically driven, which can be associated with the release of H^+^ and hydration water after coordination of Pd^2+^ cations.

**TABLE 4:** Thermodynamic parameters for the adsorption of Pd^2+^ cations on Pd‐Si‐IS and NII‐Si‐IS silica particles

	*K*_c_			−ΔG^o^_ads_ (kJ/mol)				
System	293 K	303 K	313 K	293 K	303 K	313 K	ΔH^o^_ads_ (kJ/mol)	ΔS^o^_ads_ (J/molK)
Pd‐Si‐IS	19999	24999	45453	24.12	25.51	27.91	32.21	191.63
NI‐Si‐IS	205.6	278.3	426.3	12.97	14.18	15.76	28.57	141.50

#### 3.2.3 Adsorption kinetics

**FIGURE 10:**
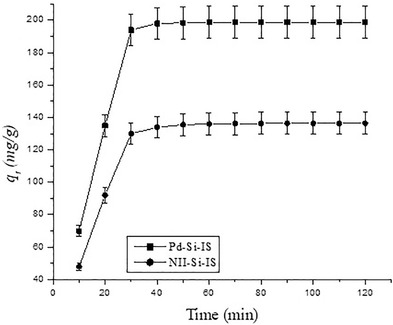
Contact time effect on removal of the Pd^2+^ cations by Pd‐Si‐IS and NII‐Si‐IS particles

Figure 10 shows the contact time effect on the adsorption of Pd^2+^ cations onto Pd‐Si‐IS and NII‐Si‐IS. The rapid initial rate of extraction from the solution may have been because of the coordination sites’ availability on the adsorbent surface. After increased contact time, the most accessible sites become saturated, which in turn can considerably decrease the adsorption rate. Both adsorbent types reach equilibrium within 1 h. To interpret the kinetic results, both pseudo‐first‐order and second‐order models were tested.^48^, ^49^

The pseudo‐first‐order model is represented by:

(9) $$ln\left(q_{e}-q_{t}\right)=\ln q_{e1}-k_{1}t$$

where *q_e_* and *q_t_* (mg/g) are the extracted Pd^2+^ cations by Pd‐Si‐IS or NII‐Si‐IS at equilibrium and any given time (*t*), respectively*. k_1_* (min^−1^) is the apparent first‐order rate constant. The first‐order parameters *k_1_* and *q_e1_* were estimated using the slope and intercept of ln*(q_e_‐q_t_)* against *t* plot, respectively.

The pseudo‐second‐order model is represented by:

(10) $$\frac{t}{q_{t}}=\frac{1}{k_{2}q_{e2}^{2}}+\frac{t}{q_{e}}$$

where *k_2_* (g mg^−1^ min^−1^) is the pseudo‐second‐order rate constant. The first‐order model parameters *k_2_* and *q_e2_* were calculated using the slope and intercept of *t/q_t_* against *t* plot.

The estimated kinetic parameters using the mathematical models above are tabulated in Table 5. The relatively higher *R* ^2^ and lower SD as long with the estimated *q_e_* values highlights that the adsorption of Pd^2+^ cations onto Pd‐Si‐IS and NII‐Si‐IS exhibited the most appropriate results using the second‐order equation, which may indicate that the metal ion coordination is the main mechanism governing the overall solid‐phase extraction procedure.

**TABLE 5:** Kinetic parameters for Pd^2+^ ions extraction by Pd‐Si‐IS and NII‐Si‐IS silica particles

Adsorbent	First‐order model		
	*k_1_* (min^−1^)	*q_e1_* (mg/g)	*R^2^*
Pd‐Si‐IS	0.112	220 ± 3	0.8765
NII‐Si‐IS	0.087	140 ± 3	0.7894

Adsorbent	Second‐order model		
	*k_2_* (g/(mg min))	*q_e2_* (mg/g)	*R^2^*
Pd‐Si‐IS	2.8 × 10^−4^	200 ± 2	0.9998
NII‐Si‐IS	1.9 × 10^−4^	136 ± 2	0.9987

#### 3.2.4 Adsorption isotherms

**FIGURE 11:**
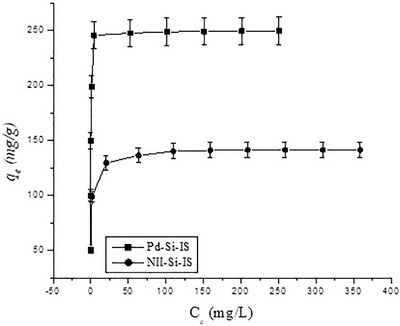
Pd^2+^ cation isotherms by Pd‐Si‐IS and NII‐Si‐IS silica particles

For investigating the adsorption capacity, several concentration experiments have been performed with variations of Pd‐Si‐IS and NII‐Si‐IS as a function of the Pd^2+^ cations’ concentration and the isotherms are illustrated in Figure 11. The capacity of Pd^2+^ ion‐imprinted and non‐ion‐imprinted silica particles amplified considerably by increasing the initial Pd^2+^ cation concentration until reaching a maximum level that is the saturation point of the active coordination sites. For more understanding and a better description of the adsorption equilibrium isotherms in aqueous solution, both Langmuir and the Freundlich models are used.

Based on the Langmuir model (Equation 11), the Pd^2+^ cations are adsorbed in form of a single layer onto energetically equivalent coordination active sites, while no interaction among adsorbed Pd^2+^ cations. The Langmuir model is presented in Equation 11.

(11) $$\frac{C_{e}}{q_{e}}=\frac{1}{q_{m}K_{L}}+\frac{C_{e}}{q_{m}}$$

where *q_e_* is the adsorption capacity (mg/g), *C_e_* is the equilibrium concentration of Pd^2+^ cations (mg/L), *q_m_* is the maximum adsorption capacity for Pd^2+^ cations (mg/g), and *K_L_* is the Langmuir adsorption constant (L/mg). The slope and intercept of the linear plot of *C_e_/q_e_* against *C_e_* were used to calculate the parameters *q_m_* and *K_L_*.

On the other hand, for a Freundlich model, the formation of an adsorbate multilayer onto energetically heterogeneous adsorption sites is expressed by:

(12) $$lnq_{e}=lnK_{F}+\frac{1}{n}\left(lnCe\right)$$

where *K_F_* and *n* are the Freundlich model parameters, which can be acquired from the slope and intercept of the linear plot of *lnq_e_* against *lnC_e_*.

The experimental isotherms of Pd^2+^ cation adsorption onto both Pd‐Si‐IS and NII‐Si‐IS are shown in Figure 11; the corresponding parameters calculated by fitting the results with the aforesaid two mathematical models are listed in Table 6. As can be noticed, for both of adsorbents, Langmuir models displayed the better fit with the equilibrium isotherm data with *R* ^2^ > 0.99, which indicated the adsorption of Pd^2+^ cations in form of single layer onto the homogeneous surface of both of the modified silica particles. The *q_m_* value corresponding to the Pd‐Si‐IS was considerably higher than the value related to NII‐Si‐IS, which can be attributed to the comparatively larger surface area by the ion‐imprinted NII‐Si‐IS as well as the formation of Pd^2+^ cations recognition hollows by imprinting procedure.

**TABLE 6:** Pd^2+^ cations adsorption parameters for Pd‐Si‐IS and NII‐Si‐IS silica particles based on different equilibrium models

	Langmuir isotherm constants		
Adsorbent	*K_L_* (L/g)	*q_m_* (mg/g)	*R^2^*
Pd‐Si‐IS	2.43	249.6	0.9987
NII‐Si‐IS	2.12	141.3	0.9897

Adsorbent	Freundlich isotherm constants		
	*K_F_*	*n*	*R^2^*
Pd‐Si‐IS	125.20	6.34	0.7689
NI‐Si‐IS	95.31	7.52	0.6897

#### 3.2.5 Adsorptive selectivity

The efficiency of selective Pd^2+^ cations recognition by both Pd‐Si‐IS and NII‐Si‐IS was evaluated. Calculated selectivity parameters are summarized in Table 7. The higher selectivity of Pd‐Si‐IS toward the target Pd^2+^ cations is obvious by the relatively higher D values compared to the other interfering ionic species. Moreover, the calculated selectivity coefficients of the target Pd^2+^ ions corresponding to Pd‐Si‐IS all have values larger than 1. Alternatively in case of NII‐Si‐IS, the projected selectivity coefficient amounts were approximately equal to or less than 1. These selectivity parameters’ amounts indicate the greater ability of the Pd^2+^ imprint sites within the network of Pd‐Si‐IS‐modified silica particles to selectively extract the target Pd^2+^ cations from multi‐ionic solutions including competing metal ions.

**TABLE 7:** Selective adsorption of target Pd^2+^ cations from multi‐ionic solutions by Pd‐Si‐IS and NII‐Si‐IS silica particles

	Distribution ratio (L/g)		Selectivity coefficient *β_Pd_^2+^_/M_^n+‏^*		
Metal	Pd‐Si‐IS	NII‐Si‐IS	Pd‐Si‐IS	NII‐Si‐IS	Relative selectivity coefficient *β_r_*
Pd^2+^	523.67	15.76	–	–	
Co^2+^	12.65	12.85	41.4	1.22	33.93
Cu^2+^	20.54	18.45	25.5	0.85	30.00
Mn^2+^	13.89	14.67	37.7	1.07	35.23
Ni^2+^	9.45	10.88	55.4	1.45	38.21

#### 3.2.6 Regeneration

The metal ions’ desorption from the adsorbent particles without adsorption efficiency loss is important for functionality of ion‐imprinted particles. In this regard, the adsorbed Pd^2+^ cations were extracted from the Pd‐Si‐IS particles via acidified thiourea solution as a desorption medium. The silica particles were then reutilized and desorbed five times in a row and each individual time, both the desorption efficiency and regeneration efficiency were calculated using Equations (8) and (9). It was noticed that the adsorption capacity marginally reduced by 5% after the fifth experiment. This might be pertaining to the partial hydrolysis of the C=N bond of the Schiff base that occurs due to continuous regeneration in acidic media.

#### 3.2.7 Conclusions

Modified Pd^2+^ ion‐imprinted hollow chelating silica particles were prepared by the reaction of AT with isatin to synthesize the polymerizable Schiff base ligand that was consequently coordinated to the template Pd^2+^. The Pd^2+^ complex solution was mixed with tetraethoxysilane and set aside to form gel under slightly basic conditions. The template Pd^2+^ cations were leached from the resultant cross‐linked matrix. The molecules and substances synthesized throughout the synthetic route were studied using appropriate physical methods to distinguish the chemical structure of the obtained chelating silica particles. Furthermore, the synthesized ion‐imprinted as well as non‐ion‐imprinted silica particles were employed in several experiments in order to optimize and examine the selective Pd^2+^ removal process.

## CONFLICT OF INTEREST

The authors declare no conflict of interest.

## Data Availability

Authors confirm that the data supporting this study are available within the article. The synthetic route is explained in full details in experimental sections in order to resynthesize the ligand and the complex. All the adsorption experiments were performed three times and the points were taken as an average. Any further information is available from the corresponding author, M.N., upon reasonable request.
